# Clinical equipoise and ethical challenges in expanding endovascular stroke thrombectomy practice

**DOI:** 10.3389/fsurg.2026.1912229

**Published:** 2026-07-22

**Authors:** Aryan Wadhwa, Evan P. McNeil, Pratham B. Bhatt, Kimberly Han, Shashvat Purohit, Justin Granstein, Christopher S. Ogilvy, Mario Ganau, Philipp Taussky

**Affiliations:** 1 Neurosurgical Service, Beth Israel Deaconess Medical Center, Harvard Medical School, Boston, MA, United States; 2 Neurosurgery Department and Neuro ITU, Oxford University Hospital, Oxford, United Kingdom

**Keywords:** clinical trials, endovascular, ethics, stroke, thrombectomy

## Abstract

**Introduction:**

Endovascular thrombectomy transformed acute ischemic stroke care while exposing ethical tensions in emergency research. As landmark trials rapidly shifted standards, investigators faced complex questions regarding equipoise, early stopping, and respecting autonomy when patients lack capacity and time is critical.

**Methods:**

We conducted a focused literature analysis from 2003 to 2024 spanning practice-changing thrombectomy trials and contemporary ethics literature. We synthesized three domains: (1) equipoise dynamics during evidence accumulation; (2) balancing non-maleficence against the social value of continuing trials; and (3) consent models for incapacitated patients in time-sensitive settings.

**Results:**

Initial uncertainty regarding endovascular efficacy justified early randomized controls. However, post-2015 data precipitated a rapid loss of equipoise, necessitating the early termination of trials to uphold the duty of care for control arm participants. While early stopping rules successfully minimized harm, they raised tensions regarding the precision of treatment effect estimates. Furthermore, the requirement for standard informed or surrogate consent was found to introduce selection bias against severe stroke patients lacking capacity. The literature supports shifting toward deferred or presumed consent models in these emergencies, arguing that rigid adherence to autonomy can paradoxically violate the principles of justice and beneficence by systematically excluding the most vulnerable patient populations from life-altering interventions.

**Conclusions:**

Ethical conduct of emergency stroke trials requires continuous reassessment of equipoise, pre-specified stopping rules that prioritize participant welfare, and consent pathways tailored to incapacity and time sensitivity. We explore principles integrating beneficence, non-maleficence, autonomy, and justice to maximize lives saved and prevent disability while preserving rights. These principles generalize to future trials as indications, technologies, and timelines evolve.

## Introduction

Acute ischemic stroke is a leading cause of morbidity and mortality, and the past 10–15 years have seen revolutionary advances in its treatment (1). Greatest among these is endovascular thrombectomy for large-vessel occlusion stroke, even with large core infarcts, which multiple trials since 2015 have shown to improve patient outcomes when added to standard care (2, 3). These thrombectomy trials–including MR CLEAN, ESCAPE, REVASCAT, SWIFT PRIME, DAWN, DEFUSE 3, and others–have not only transformed clinical practice, but also provoked important ethical questions about the conduct of randomized clinical trials in emergency settings (2–6). Given the critical involvement of endovascular neurosurgeons and neurointerventionalists in endovascular stroke treatment, we explore key ethical questions pertinent to neurosurgical care teams that emerged during these trials: equipoise and randomization, trial continuation, informed consent, and patient autonomy.

### Background: thrombectomy trials and their impact over the past 15 years

In the early 2010s, several randomized trials (IMS III, SYNTHESIS Expansion, MR RESCUE) failed to show a long-term clinical benefit of endovascular therapy over intravenous thrombolysis, casting initial doubt on the intervention (7–9). However, these studies were later recognized as limited by older endovascular technology and broad inclusion criteria (10). Thrombectomy was brought back to the foreground with the MR CLEAN trial in the Netherlands, which published positive results in 2014 (2). MR CLEAN was a multicenter trial of 500 patients that for the first time demonstrated a significant benefit of endovascular thrombectomy in acute stroke. Patients randomized to thrombectomy plus medical therapy had substantially better functional outcomes at 90 days than those receiving medical therapy alone, without increased mortality. This was a pivotal moment: the first major trial establishing endovascular treatment as effective and safe within 6 h of stroke onset.

**Figure 1:**
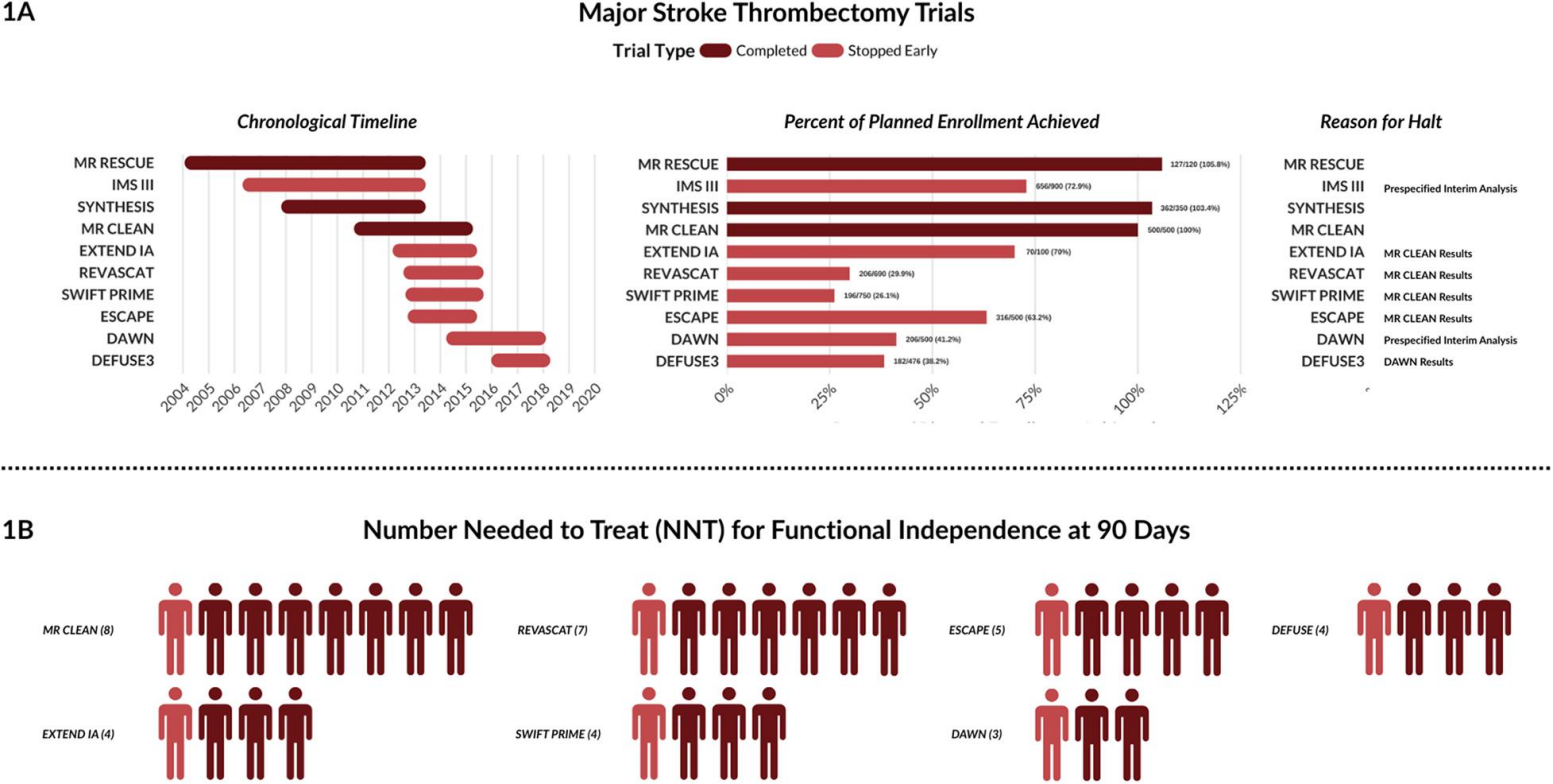
**(A)** Major stroke thrombectomy trials from 2004 to 2020, represented from first enrollment date to first published results date. **(B)** Number needed to treat (NNT) for functional independence at 90 days as demonstrated by key thrombectomy stroke trials.

In early 2015, several other trials, including ESCAPE, EXTEND-IA, SWIFT PRIME, and REVASCAT, were already in progress internationally to test thrombectomy (3–5, 11). Considering MR CLEAN's positive findings, these studies performed unplanned interim analyses and found that their efficacy endpoints had been met early. One by one, the trials were halted ahead of schedule due to clear benefit favoring thrombectomy. By mid-2015, scientific consensus emerged, and U.S. guidelines recommended thrombectomy as standard care (Figure 1A) (12).

A second wave of thrombectomy trials around 2018 (e.g., DAWN and DEFUSE 3 in the U.S.) extended the treatment time window out to 16–24 h for selected patients (6, 13). By then, standard therapy up to 6 h was firmly established, so these new trials enrolled only patients presenting later or with advanced imaging selection, again restoring a degree of uncertainty for the question of late intervention. Those trials were again stopped early for efficacy when interim analyses showed significantly better outcomes with thrombectomy in the extended window, again raising questions about balancing prompt trial termination against full data collection.

## Discussion

### Defining equipoise in stroke trials: when does randomization become unethical?

The rapid succession of positive trials in 2015 raises the first ethical issue of equipoise. Equipoise refers to a state of genuine uncertainty in the expert medical community about which trial arm is superior (14, 15). Equipoise is an ethical prerequisite for randomizing patients: if one treatment is known to be better, it would be unethical to deny it to half the participants.

At the start of the thrombectomy trials, clinical equipoise clearly existed: prior studies had been neutral or negative, and the medical community was unsure of endovascular intervention's net effect in acute ischemic stroke. Under those conditions, enrolling patients into a randomized comparison of thrombectomy vs. medical therapy was ethically sound and offered the prospect of important new knowledge.

Once MR CLEAN's results became known, continuing to randomize patients to a non-thrombectomy arm came under ethical scrutiny. Many stroke physicians were convinced the case was already clear: endovascular intervention saves lives and prevents disability. From their perspective, equipoise had been lost; no reasonable clinician could any longer be truly unsure that thrombectomy was beneficial in appropriate patients. If that were so, then any further randomization of patients to a control arm denying them thrombectomy might constitute a breach of the physician's duty of care, violating beneficence and non-maleficence.

On the other hand, other experts urged caution, arguing that one trial alone was not sufficient to entirely eliminate uncertainty. In an editorial accompanying MR CLEAN, Werner Hacke stated: “It is premature to conclude that there is no longer equipoise regarding thrombectomy. We need and will get results from other well-designed trials, not only to confirm or refute the results of MR CLEAN but also to look at effects in subgroups.” (16) The initial evidence, while compelling, had limitations; MR CLEAN was done in a single country with specific protocols and inclusion criteria. The ongoing trials (ESCAPE, EXTEND-IA, etc.) could provide confirmatory data across more diverse healthcare systems and answer questions about variations in stroke severity, occlusion anatomy, imaging criteria, and time to treatment. This tension surely persisted among decisions by investigators and data monitoring committees about whether and when to stop the trials early.

The literature suggests a tipping point came rapidly, however, as multiple trials all started reporting early positive results (Figure 1A). By April 2015, when ESCAPE, SWIFT PRIME, REVASCAT and others announced their findings, the aggregate evidence was overwhelming. Clinical equipoise for the question “Should an acute stroke patient with a large-vessel occlusion get thrombectomy within 6 h?” had definitively vanished. It was no longer ethically acceptable to randomize such patients to medical therapy alone in further studies. The remaining uncertainties shifted to new questions (e.g., how late one can treat, how to treat patients with large established infarcts or milder strokes), which spawned new trials where equipoise existed for those specific scenarios. A similar ethical transition is now occurring with medium-vessel occlusion (MeVO) stroke. Unlike proximal large-vessel occlusion, where thrombectomy is now established standard care, the benefit of endovascular therapy for MeVO remains under active investigation, restoring genuine clinical equipoise and justifying contemporary randomized trials (17). Accordingly, the ethical challenges surrounding consent, randomization, and trial continuation in MeVO more closely resemble those encountered during the early large-vessel thrombectomy trials than current LVO practice. This dynamic illustrates how equipoise is time-sensitive and evidence-dependent: a trial can begin in a state of uncertainty and lose that uncertainty partway through due to external developments or internal interim results. Ethical research requires continuous monitoring of equipoise; investigators and data safety monitoring boards (DSMBs) must be prepared to stop or modify trials if the uncertainty requirement is no longer met.

In the context of stroke and thrombectomy, largely agreed upon as lifesaving interventions, one could argue that the ethical threshold for equipoise is narrower: clinicians tolerate less uncertainty when decision-making risks severe morbidity or mortality. The moral duty to rescue largely competes with scientific caution; in other words, the degree of evidence needed to justify offering a potentially life-saving intervention may be lower than other contexts. While possibly controversial, mechanical thrombectomy in its early era may not have necessarily been considered “lifesaving” as much as “disability-sparing,” given patients were likely to survive either way but with vastly different levels of neurological status. As such, equipoise threshold likely continued to remain higher, as opposed to emergent procedures.

Another interesting perspective to consider is the need for randomized control groups, rather than historical controls, in the context of stroke. Major interventions like percutaneous coronary intervention (PCI) never underwent rigid RCTs, prompting questions about why stroke required them (18). Historical controls can be deemed ethically and scientifically acceptable largely when the disease presentation, diagnostic accuracy, and standard of care remain stable across time. In the situation of acute stroke, this was not the case: rapidly evolving upgrades in imaging, workflow efficiency, and thrombectomy devices could hence make historical comparisons unreliable, and consequently equipoise present. Unlike PCI, where pathophysiologic mechanisms and direct reperfusion could be visualized and rapidly verified, early thrombectomy trials faced uncertainty in patient selection, device efficacy, and imaging selections. In these cases, randomization remained essential to distinguish procedural characteristics from true therapeutic effect. By the early 2010s, intravenous tPA had become the gold standard for reperfusion therapy within 4.5 h of stroke onset. However, the incremental value of endovascular thrombectomy complementary to tPA remained uncertain, further clouded by the heterogeneous results of early trials that had failed to demonstrate superiority. This uncertainty largely justified continued randomization to tPA-only control arms rather than just relying on historical controls in this situation.

Only once MR CLEAN had completely resolved that uncertainty, through robust consistent evidence via a rigid RCT, that thrombectomy was superior to tPA alone, could this equipoise be lost in stroke. The need for RCTs in stroke thus stemmed not from rigid methodological orthodoxy, but from genuine uncertainty in which subgroups derived benefit—a central ethical requirement of equipoise.

### Continuing trials vs. early stopping: additional deaths for long-term certainty?

One of the starkest ethical questions during the thrombectomy trials was whether it was justifiable to allow additional patients to have bad outcomes–including death–in the control arm to accrue a larger sample size and more robust evidence (19). This is essentially a conflict between the immediate duty to minimize harm to current research participants (honoring non-maleficence and beneficence for those individuals) and the longer-term goal of generating generalizable knowledge that could benefit many future patients (a utilitarian or social beneficence argument). The literature on research ethics recognizes this tension. When an experimental treatment shows clear benefit early, continuing the trial means knowingly withholding a life-saving treatment from those randomized to the control, a profound moral dilemma (20).

Non-maleficence (“do no harm”) weighs heavily here: researchers have an ethical obligation not to harm participants, and once a treatment's benefit is established, withholding it can be construed as causing harm. This perspective aligns with the duty to rescue: the moral duty of a physician to save a life or prevent serious harm when possible. In an ongoing trial, once it becomes evident that one arm offers rescue and the other does not, physicians on the DSMB (and treating clinicians at trial sites) face the duty to rescue those in the inferior arm by stopping the trial or switching them to the better therapy (Figure 1B). This is standard practice via interim analysis.

However, ethicists and trialists also highlight contrary arguments for why stopping every trial at the first sign of benefit might not always be ideal. Scientifically, an abbreviated trial can overestimate the treatment effect or fail to capture longer-term outcomes (20). A systematic review by Montori et al. demonstrated that trials stopped early for benefit often exaggerate the magnitude of benefit compared to later, larger studies (21). Thus, from a beneficence to future patients standpoint, there exists risk that prematurely stopping a trial could lead to implementing a therapy based on incomplete evidence, possibly misleading practice. Ethically, this raises the concept of justice as well. Future patients (and society) deserve reliable, well-powered evidence on which to base treatment decisions. If a trial is stopped with an insufficient number of patients because of a dramatic early trend, the resulting knowledge might be less precise about which subgroups benefit or the true magnitude of effect. The ethical principle of justice, in the context of research, includes fairness to future patients and a duty to generate knowledge that is generalizable and applicable to all groups who may need the treatment. Stopping too soon could shortchange this goal.

To directly address the question: “*Is it ethical to allow additional patient deaths to attain greater statistical power?*”, the prevailing view in literature and practice is no, not beyond the minimum needed to be reasonably sure of the result (22). In ethical terms, this decision aligns with beneficence and non-maleficence: beneficence toward future patients is served by obtaining solid evidence up to a point, but non-maleficence toward current subjects sets a firm limit; that point where evidence is “good enough” must be defined in advance (via interim stopping rules) and respected. In the thrombectomy trials, the DSMBs' actions reflected this principle. Each of the major 2015 trials was halted as soon as interim data convincingly demonstrated benefit, thereby minimizing further harm to controls. Investigators thus attempt to honor both present and future lives by using group sequential designs that stop trials at an optimal ethical endpoint.

### Informed consent and patient autonomy

Autonomy—the right of patients to make informed decisions about their own care—is a foundational principle of medical ethics and is embedded in research regulations requiring informed consent of participants (23). Yet acute stroke trials present a unique scenario where obtaining informed consent is often impractical or impossible in the high-stakes moment (24). A major stroke frequently impairs consciousness, language, or cognition, leaving the patient incapable of making decisions at the very time when treatment decisions (and trial enrollment decisions) must be made within minutes. This sparked extensive discussion on conducting ethical stroke trials while respecting autonomy under these constraints.

In stroke trials, surrogate consent is the common solution, upholding autonomy indirectly by assuming the surrogate decides as the patient would have (25). However, this is an imperfect proxy for true informed consent, and ethical concerns remain about whether surrogates can adequately represent the patient's wishes in a trial setting. This contrasts with pediatric emergency practice, where guardian consent is generally required before invasive procedures, contributing to treatment delays and limiting the feasibility of direct-to-angio pathways and randomized trials in children. Consequently, pediatric evidence is derived largely from observational studies and selective extrapolation from adult data (26). Despite these issues, surrogate consent is generally accepted by ethics guidelines for acute interventions when patient consent is unattainable, provided the risk is not excessive.

Challenges arise when surrogates are not immediately reachable. The pathophysiology of stroke inherently requires treatment within a narrow therapeutic window, and in many cases, patients arrive to the hospital alone and locating a decision-maker in time can be infeasible. One analysis found that patients able to consent were often less severe strokes, whereas those unable (requiring surrogate) were more severe and older (27, 28). Consequently, trials that only included patients with capacity or available surrogates ended up systematically excluding some of the sickest patients, a selection bias with ethical and scientific implications. A study by Hotter et al. observed that patients who lacked consent capacity and thus often got excluded were more likely to have worse strokes (aphasia, higher National Institutes of Health Stroke Scale) and different outcomes (29). The authors concluded that if outcome measures of a trial are affected by this selection bias, it appears unethical to exclude patients unable to consent. In other words, justice and scientific validity demand that we find ways to include those patients, rather than simply excluding a vulnerable subset and then generalizing results inappropriately. Excluding those who cannot consent might also disproportionately exclude older adults who are less likely to have immediate surrogates or limited English proficiency families, raising justice concerns about fair access to research and potential benefits.

A 2003 editorial argued that stroke researchers should consider conducting trials with an exception from the informed consent requirement in select situations (30). More recently, the Canadian AcT trial (comparing tenecteplase vs. alteplase thrombolysis) was designed with a deferral of consent model, and ethicists provided justification that deferred consent can be acceptable and even ethically superior to surrogate consent in some cases (31). The rationale was that deferred consent can reduce bias and improve inclusivity—for example, enrolling the sickest patients who lack capacity, then obtaining consent from them or families later, avoids systematically excluding those patients. It also speeds up treatment (or “door-to-puncture” time) by not waiting for lengthy consent discussions in the emergency department, thereby honoring the principle of beneficence (maximizing the patient's chance of a good outcome). Of course, deferred or waived consent carries ethical risks, primarily that it compromises patient autonomy: enrolling someone in research without their documented permission. Patients may be enrolled into trials “against their wishes” if later they or their family say they would not have wanted experimental treatment. This is a serious concern as it could undermine public trust and violate the individual's rights.

In recent guidance, the American Academy of Neurology (AAN) issued a 2022 position statement addressing consent in acute stroke care and research (32). The AAN affirmed that when patients lack capacity and no surrogate is available, presumed consent to treatment is acceptable for proven therapies: physicians may treat emergently (for example with thrombolysis or thrombectomy) on the presumption that a reasonable person would consent to a potentially life-saving intervention in an emergency. This is consistent with standard emergency care doctrine.

In practice, many stroke trials have navigated consent by using a mix of approaches: urgent surrogate consent if available, enrollment of only those with consent (leading to bias), or waivers for very minimal risk studies. There is increasing recognition that innovative consent models are needed to conduct timely research without undermining patient rights. Some proposals include simplified consent forms for emergency research, video consent delivered en route by paramedics (as tried in the FAST-MAG prehospital stroke trial), or community consent with patient opt-out options (33). The goal is to uphold respect for patients as much as possible while not sacrificing beneficence. Investigators in one stroke trial (AbESTT-II) reported feeling that obtaining full informed consent produces unnecessary delays yet felt a full waiver was inappropriate, highlighting the dilemma (34). This exemplifies the conflict between autonomy and beneficence: we always want to respect the patient's right to choose, but we also want to do what is best for them medically in a time-sensitive situation.

In summary, the literature suggests a nuanced approach, highlighting some common principles. Transparency with the community and after-the-fact consent can help uphold respect for persons. Autonomy is not an on/off switch but a spectrum in acute care—we strive to maximize it, but some temporary suspension of autonomous choice can be ethically permissible in emergencies to serve the patient's own interests or the greater good, provided other protections are in place. The ongoing dialogue between stroke researchers and ethicists aims to refine these practices so that future acute stroke trials can proceed both ethically and efficiently.

## Conclusions

The past decade of thrombectomy trials in acute ischemic stroke has provided not only clinical breakthroughs but also rich lessons in research ethics. This literature review finds that maintaining ethical integrity in such trials required continuous re-assessment of equipoise and a willingness to make difficult decisions, such as stopping trials early to avoid harm once benefit was evident or modifying the time-of-intervention consent process to maximize beneficence. The ethical consensus is that patients should not be treated as mere means to an end; any sacrifice asked of them (such as being randomized to medical management only) must be justified by genuine scientific uncertainty. When that uncertainty is resolved, the ethical balance shifts decisively toward delivering the highest quality intervention to the highest number of people as expeditiously as possible.
